# Device Therapy to Prevent Sudden Death in Patients with Structural Heart Disease

**Published:** 2010-06-05

**Authors:** N Sreeram, U Trieschmann, M Emmel, M Khalil

**Affiliations:** 1 Department of Paediatric Cardiology. University Hospital of Cologne, Germany; 2 Paediatric Cardiac Intensive Care and Emergency Medicine. University Hospital of Cologne, Germany

**Keywords:** sudden cardiac death, device therapy, structural heart disease

## Abstract

The advent of the implantable cardioverter defibrillator has provided clinicians with a potential tool to prevent sudden arrhythmic death. When considering patients with structural heart disease, long-term follow-up data have suggested that this is indeed an important cause of late mortality. It is essential therefore to undertake follow-up studies to identify high risk individuals or disease categories that are associated with sudden cardiac death (SCD), and to elucidate the specific risk factors that may be associated with this complication. We provide a brief update on the current state of knowledge in this challenging and rapidly developing field.

## Introduction

The introduction and increasingly widespread use of implantable cardioverter defibrillators (ICDs) has potentially provided us with a tool to prevent sudden arrhythmic death. While their use in specific subsets of adult patients with structural or functional heart disease has been established, their role in young patients with structural heart disease, the majority of whom have undergone surgical procedures leading to definitive repair or permanent long-term palliation is still being defined. The results of surgical repair of structural heart disease have continued to improve with time, to the extent that survival after surgery, even for complex defects, is almost taken for granted. This in turn means that there is a growing population of adolescents and young adult survivors, a significant proportion of whom will require life-long follow-up. In addition to new or residual haemodynamic sequelae, either as part of the natural history or the post-surgical history of the original malformation, additional findings during follow-up include progressive ventricular failure, and the occurrence of clinically significant arrhythmias, some of which may be associated with a risk of sudden cardiac death. It is incumbent on practising paediatric cardiologists to critically evaluate the role of ICDs in this population, in order to establish definitive guidelines for implanting such devices, with a view to assessing the benefits (in terms of saving lives) versus the potential risks, alterations in lifestyle and quality of life of the individual patient in whom an ICD has been implanted, and the cost versus benefit ratio. Current strategies for preventing sudden cardiac death(SCD)  rely on risk assessment for individual patients or specific disease categories, and implantation of ICDs in patients considered to be at high risk. In population terms however, the absolute number of SCDs that occur in the general (relatively low risk) population is large when compared to the number of SCDs in a well defined high risk population. This is true for both adult and paediatric populations [1,2]. Therefore, prevention of sudden cardiac death in the high risk population is unlikely to prevent the majority of SCDs. Another important caveat is that although ICD therapy may be cost-effective for individual patients, they may nonetheless be too costly for most worldwide societies, even if surgical facilties for repairing structural heart defects may be in place. Risk stratification therefore also means identifying subsets of patients in whom ICD implantation will not be useful. The following is a summary of our current knowledge, with a personal viewpoint on the indications for ICD implantation in young patients with structural heart disease.

## Defining the risk for sudden death in young patients

In 1996 Maron and colleagues addressed the issue of sudden death in 134 young athletes aged <40 years in a landmark paper [3]. Apart from congenital coronary artery anomalies, the majority of which may not present with symptoms prior to the occurrence of an adverse ischemic event, aortic valve stenosis was the only other congenital structural defect which was associated with sudden death in this select population. Population based studies from both Israel and Italy, involving young individuals (non-athletes) have substantiated the findings of Maron et al [4,5]. Based on such population based studies, it would be safe to state that congenital structural heart disease, particularly following surgical repair at a young age, does not account for a significant proportion of sudden deaths. To further test this assumption, a nationwide study was carried out in the Netherlands [6]. From the hospital records, and after contacting the responsible paediatricians and if necessary, the general practitioners, all children who had died suddenly during a 11 year period were identified. This was possible in practice because the Netherlands has a well defined and stable population (of around 16 million inhabitants), health insurance is compulsory for all patients, and there is a limited number of centers which care for children with structural heart disease. For the purpose of identifying all patients who would fit into the diagnostic category of sudden death, the definition of sudden unexpected death was modified to mean death within 24 hours of onset of the precipitating symptom which required emergency hospital admission or led to death prior to hospitalization, in patients who were otherwise not expected to die (the obvious exclusion criterion was patients with terminal heart failure or awaiting transplantation). A total of 150 sudden unexpected deaths were identified, 101 of whom were above one year of age. Although the majority of these patients had a cardiac cause for sudden death, arrhythmia was only one of several causes of death.

On the other hand, the risk of late sudden death for patients who survive surgery for congenital heart defects is considerably greater than for an age-matched control population. The risk of sudden death appears to be time dependent, and increases primarily after the second postoperative decade [7-9]. This means that it is not sufficient to confine ourselves to a study of the paediatric population, but to extend follow-up into adult life. The majority of late sudden deaths appear to be arrhythmic, and children and young adults with structural heart disease are likely to have ventricular arrhythmia or pulseless ventricular tachycardia as the presenting arrhythmia during out of hospital cardiac arrest, in contrast to younger children in whom asystole or pulseless electrical activity is more common [2,10].

Finally, it is also necessary to take into account changes in the post-surgical history of specific subsets of patients, particularly those with complex single ventricle physiology undergoing staged Fontan palliation, for whom long-term follow-up data were not available in some of the earlier published studies. Based therefore on currently available data, four categories of patients with structural heart disease appear to have the highest risk of sudden arrhythmic death during follow-up: tetralogy of Fallot following surgical repair; transposition of the great arteries following an atrial (Mustard or Senning) repair; congenital aortic valve stenosis (and other left ventricular outflow tract obstruction variants amenable to surgical or catheter-based palliation; hypertrophic obstructive cardioymopathy will not be considered here as it falls into a separate risk category) and single ventricle physiology following Fontan type of palliation.

## Tetralogy of Fallot

### Identifying the patient at risk of SCD

Sudden cardiac death has been reported to be the most important cause of mortality following repair of tetralogy of Fallot, with an annual mortality rate, from population based studies, of 0.3% [7]. A wide variety of risk factors have been associated with late SCD in tetralogy of Fallot. Some of these include older age at repair, early transient heart block, longer duration of clinical follow-up, the presence of a right ventricular outflow tract patch, and high grade ventricular ectopy [11-13]. Electrocardiographic markers for ventricular arrhythmia and SCD include QRS duration ≥180 ms, the annual change in QRS duration during follow-up (in msec/year), and increased QT and JT dispersion [14-16]. The ECG markers have in turn been believed to reflect the degree of right ventricular enlargement (due to progressive pulmonary valve insufficiency with consequent right ventricular volume overload) and right ventricular scarring as a consequence of chronic stretch, ischemia and surgical incisions. While a QRS duration of ≥180 ms has been shown to be highly sensitive in predicting patients at risk of ventricular arrhythmia, it is clear that not every asymptomatic patient with prolonged QRS duration is a candidate for an ICD. This has led to the use of invasive electrophysiologic studies (EPS) to further identify high risk patients. The first of these large scale studies involved 130 patients from all four risk categories (tetralogy of Fallot, transposition of the great arteries following atrial repair, left ventricular outflow tract variants and univentricular hearts) [17]. The majority of patients had either had a documented ventricular arrhythmia, resuscitated sudden cardiac arrest, or presented with symptoms of palpitations or syncope. In this highly selected population a positive (arrhythmia inducible) ventricular stimulation study was highly sensitive in predicting future life-threatening arrhythmic events. Unfortunately however, a negative (arrhythmia non-inducible) EP study did not mean a low risk of future arrhythmic/SCD events. A subsequent invasive EPS study confined only to tetralogy of Fallot patients was carried out by Khairy et al [18]. In 252 unselected (in 36% of the patient population, EPS was performed not for a strong clinical indication, but as part of routine screening) patients, while the positive predictive value was rather low (55%), the negative predictive value (arrhythmia non-inducibility correlating with absence of arrhythmic events at follow-up) was encouragingly high (91%). Induction of monomorphic or polymorphic ventricular tachycardia during EPS were predictors of future arrhythmic events. Risk factors for inducible ventricular tachyarrhythmia during EPS include older age at EPS, palpitations, prior palliative surgery, high grade ventricular ectopy on Holter electrograms, and a cardiothoracic ratio on chest x ray of ≥ 0.6. While the majority of earlier studies focused on the association between right ventricular dysfunction and the risk of sudden death, more recent studies have emphasised the importance of left ventricular dysfunction in predicting SCD, with the highest predictive values (positive and negative) being achieved using a combination of QRS duration and indices of left ventricular dysfunction [19].

### ICDs in tetralogy of Fallot

Two multi-centre studies to date have focused on the efficacy of ICD therapy in tetralogy of Fallot [20,21]. Khairy and colleagues reported on the results of ICD implantation in 121 adult patients (mean age 32 years, median follow-up of 3.7 years); 68 patients had received a device for primary prevention compared with 53 for secondary prevention. As may be expected, freedom from appropriate ICD discharges was higher for the primary prevention group at all time points (85% versus 79% at 1 year, 80% versus 66% at 2 years, and 67% versus 54% at 5 years post-implant). Predictors for appropriate ICD discharge were also different in the two groups, with syncope, inducible sustained polymorphic ventricular tachycardia and prior use of a class 1A or 1C antiarrhythmic drug at hospital discharge predicting an appropriate discharge in the secondary prevention group and a high LV end-diastolic pressure being the only independent predictor for appropriate ICD discharge in the primary prevention group [20]. Yap et al reported on their experience with ICD implantation in adults with structural heart disease in the Netherlands, with the majority of their patients (40 of 64 patients in the study) having undergone repair of tetralogy of Fallot [21]. The majority of the patients had undergone ICD implantation for secondary prevention, having presented with symptoms ranging from aborted cardiac arrest, spontaneous sustained ventricular tachycardia, syncope or palpitations. In contrast to the Khairy study however, during the entire period of follow-up 160 of 204 shocks in the study population were inappropriate. The only predictor for an inappropriate shock was the diagnosis of tetralogy of Fallot. There were a total of 3 possibly life-saving shocks in the tetralogy group, and it was unclear whether all shocks had been delivered in the same patient. This points to possible overuse of ICDs in this population, and raises further questions about appropriate risk stratification. Unfortunately, it is also not possible when deciding to implant an ICD in an individual patient to distinguish between appropriate versus life-saving shocks. It has been proposed therefore to adopt a scoring system, by assigning points to specific risk factors which include age, technique of surgery, heart size on chest x ray, ECG markers such as QRS duration and invasive measurements of LV end-diastolic pressure [22]. Again, although many of these markers indirectly reflect right ventricular volume overload, MRI calculations of right ventricular volumes have not been as yet included in such algorithms. On the basis of an individual patient's risk, one could then reasonably subdivide patients into one of 3 categories of risk (low=conservative therapy; high=SCD prevention measures including ICD implantation, intermediate=further risk assessment by invasive EPS and then moving the patient to either a high or low risk category) [22]. Future studies will have to determine the efficacy of such an approach in the prevention of SCD.

### Other therapeutic approaches to preventing sudden death in tetralogy of Fallot

#### Pulmonary valve replacement

Timely pulmonary valve replacement (based in some instances on right ventricular volume calculations from MRI, is being aggressively carried out in several centers. The possibility of  percutaneous valve implantation in selected patients with a pre-existing right ventricle to pulmonary artery conduit has also made this therapeutic option attractive. Achieving pulmonary valve competence would in theory relieve right ventricular volume overload, and it was hoped that this would in turn reduce arrhythmia propensity. The earliest study to look into this issue suggested that pulmonary valve replacement, combined with intraoperative cryoablation in selected patients might indeed reduce the incidence of ventricular arrhythmias [23]. In 70 patients who underwent pulmonary valve replacement (introperative cryoablation was performed in 9 patients) there was a decrease in the incidence of ventricular arrhythmias at follow-up (mean duration of follow-up was 4.7 years) from 22% to 9%; there was however a perioperative mortality of 4%. Subsequent studies have looked at the effect of valve replacement not only on arrhythmia propensity, but also the incidence of ventricular arrhythmia or sudden death during follow-up [24,25]. Oosterhof et al demonstrated that late pulmonary valve replacement resulted in a transient decrease in QRS duration, but without long-term effect. The risk of recurrent ventricular tachycardia remained substantial when the preoperative QRS duration was ≥ 180 ms, and there was no significant difference in the number of patients reaching a composite end point of VT/SCD/appropriate ICD discharge when comparing those with a preoperative QRS duration of between 150-180 ms versus those with QRS duration ≥ 180 ms [24]. Harrild et al performed a matched comparison between 2 similar patient subgroups, one of which underwent pulmonary valve replacement for symptomatic pulmonary regurgitation. Again, there was no change in QRS duration as a result of valve replacement. Valve replacement also did not reduce the incidence of ventricular arrhythmia or death at follow-up; in addition intraoperative cryoablationdid not cure ventricular tachyarrhythmia in 5 of 7 patients in whom this was performed [25].

#### Catheter ablation of sustained ventricular tachycardia and cardiac resynchronisation

There is as yet no evidence that catheter ablation of reproducible, clinically relevant VTs can abort sudden cardiac death. It is our practice, and that of others, that patients with haemodynamically poorly tolerated ventricular tachyarrhythmias receive an ICD, even if the arrhythmia substrate has been successfully ablated [26]. There are anecdotal reports of cardiac resynchronisation resulting in a decrease in ventricular size and improved cardiac output. Whether this translates to a reduced risk of sudden death is unknown.

To summarise therefore, the risk of SCD remains low, following correction of tetralogy of Fallot, and this is particularly so in the first 15 years after surgery. In patients without documented polymorphic VT/resuscitated SCD, no single variable (clinical, ECG, haemodynamic or EPS) is adequate to guide management decisions, and hence a combination of several of these markers, in combination with invasive EPS will be required to assess risk. Pulmonary valve replacement, with or without catheter or intraoperative ablation of VT substrates does not appear to attenuate the risk of future VT or SCD. Indications for ICD implantation for primary prevention of SCD remain to be established.

## Transposition of the great arteries following Mustard or Senning repair

Sudden death has been reported to be the most common cause of death following atrial repair of TGA [27-30]. Predictors of mortality include impaired systemic ventricular function, advanced NYHA class and pulmonary hypertension. While progressive sinus node dysfunction and recurrent intraatrial re-entrant tachycardia have been commonly seen with increasing duration of follow-up, the causal role of such arrhythmias in sudden death is as yet not established, although a Finnish report suggested that the loss of sinus rhythm and increased QT dispersion were risk factors for late sudden death [31]. The authors however matched 22 sudden death patients with 24 randomly chosen age-matched controls without arrhythmia or heart failure. Kammeraad et al looked specifically into the risk factors associated with sudden death, in a case control study from a single country, the Netherlands. All SCD patients were matched with two controls of the same age, who had had a similar operation at the same center, and performed by the same surgeon within the same time-frame as the index SCD patient [29]. Risk factors for SCD were the presence of symptoms of either tachyarrhythmia or heart failure, or a documented atrial tachyarrhythmia. Medications (antifailure or antiarrhythmic) or permanent pacing were shown not to be protective. There was insufficient data to assess whether catheter ablation of atrial re-entrant tachyarrhythmias was protective. A subsequent report by Dos et at also confirmed that advanced NYHA class and a history of atrial tachyarrhythmias were independent predictive factors for late mortality [30]. Of particular interest in the Kammeraad study was the finding that where a terminal  rhythm was recorded, it was almost invariably ventricular tachycardia or fibrillation. A more recent study has indeed demonstrated that ventricular arrhythmias are not uncommon in these patients [32]. The risk factors for ventricular arrhythmias and sudden death included advanced NYHA class, systemic ventricular dysfunction, a QRS duration ≥ 140 ms, and the presence of associated lesions. In contrast to the previously reported data, although atrial tachyarrhythmias were more common in their adult population, these did not seem to predict the risk for VT or sudden death.

### ICDs in the Mustard/Senning population

 Apart from anecdotal data, a single multi-center study (37 patients from 7 centers enrolled)  has investigated the outcome of ICD implantation in this population [33]. In 50% of patients receiving an appropriate shock, a supraventricular tachyarrhythmia either preceded or coexisted with VT in 50% of patients, suggesting that rapid conduction of supraventricular arrhythmia may be causal for VT. Annual rates of appropriate shocks when an ICD had been implanted for primary prevention indications were 0.5%. The predictors for an appropriate shock were a secondary prevention indication for ICD implantation, and the absence of beta-blockers. These findings together suggest that there is no established role for ICD implantation as primary prevention of SCD, that supraventricular arrhythmias need to be aggressively treated (by catheter ablation whenever possible), and that routine beta-blocker therapy is recommended for this patient population. Prior EPS and inducibility did not predict which patients would receive an appropriate shock (although the majority of patients in the multi-center study had received an ICD for primary prevention), and the incidence of generator and lead-related complications was high, suggesting that we should be very selective in choosing candidates for ICD implantation. 

## Late deaths after the Fontan procedure

Sudden death is one (and probably not the most important) of several possible mechanisms for late mortality after the Fontan procedure [34]. ICD implantation in this patient group should therefore be reserved for secondary prevention, given the current state of knowledge.

## Left ventricular outflow tract obstruction

Several follow-up studies have confirmed that the long-term survival of patients with coarctation of the aorta is less than may be expected for what is an extracardiac lesion [35-38]. Factors that have been implicated as causes of death include later age at surgery (and consequent persistent systemic hypertension), residual or recoarctation, persistent systemic hypertension causing left ventricular hypertrophy, early coronary artery disease, rupture of intracranial aneurysms, and sudden death. Aortic valve and aortic arch pathologies are also more commonly encountered in patients with repaired coarctation, necessitating careful follow-up and early reintervention [39]. The treatment strategies should therefore include early primary repair, careful follow-up and adequate blood pressure control, early detection of residual obstruction and prompt therapy of the same. Even minor degrees of residual coarctation can result in left ventricular diastolic dysfunction, in persistent hypertension and vascular damage, and it is well known from adult studies that left ventricular hypertrophy is by itself an independent factor for predicting death [40,41]. ICD therapy in this setting should only be for secondary prevention, after all predisposing causes for persistent hypertension and LVH have been adequately addressed.

## Conclusions

Early diagnosis, optimal pre-and postoperative care and improved surgical techniques have all combined to reduce the operative mortality for the entire spectrum of structural heart defects. The emphasis has therefore shifted from operative survival to issues such as late complications, natural (or "unnatural" post-surgical) disease progression, identifying and treating residual defects or new disease manifestations, improving the quality of life, and identifying and treating the risk factors associated with sudden cardiac death. Even in large nationwide studies, it has been demonstrated that late survival after cardiac surgery is lower than for the general population [42]. The majority of patients die from the sequelae of their structural heart defect, and sudden death accounts for a relatively small percentage of late mortality. While targeted therapy may be effective in selected patients, the majority of patients at risk of sudden death will probably require hybrid therapeutic strategies consisting of correction of residual adverse haemodynamic sequelae, surgical or catheter ablation of ventricular and atrial arrhythmia substrates, appropriate pharmacologic therapy for arrhythmias or heart failure, cardiac resynchronisation therapy and ICD implantation. Risk assessment for SCD in this diverse patient population remains a challenge due to the heterogeneity of structural heart disease, the low annual mortality rates and the unpredictable timing of events.
